# Hydatids in the Ventricles of the Brain

**Published:** 1849-07

**Authors:** L. Granger

**Affiliations:** Lawrenceville


ART. VI.—*Hydatids in the Ventricles of the Brain.* Communicated in a Letter to Dr. Lee, by L. Granger, M. D.

Nathan Gillett, aged about 60 years, of sanguine temperament, and rather inactive habits, but of a good general constitution, had for several years been subject to occasional attacks of severe pain in the head, for which he did not apply for medical treatment; had in the early part of January, 1845, an apoplectic attack, which produced a partial prostration of the intellect, and slight though, temporary paralysis of the limb and arm of the right side. He recovered, though slowly, the use of the limbs, but he intellect remained so far disturbed as to lead those who saw him to suppose he might be intoxicated. He soon became subject to paroxysms of furious mania, and personal restraint, finally, became necessary. He was taken to the Lunatic Asylum at Utica, where he remained a short time. He was then taken to Doct. White's private Asylum, at Hudson, where he remained for a few weeks under treatment, when he was pronounced incurable. He was brought home, and placed in a secure, well-ventilated room, where he was as comfortable as wealth and anxious friends could make him. He soon sunk into a state of perfect idiocy, relieved only by occasional paroxysms of furious and boisterous mania. He kept himself continually in a squatting posture for more than two years, in defiance of every effort of his friends, so that at the time of his death, adhesions at the hip and knee joints were found, of such strength that the effort to straighten the body enough to place it in an ordinary coffin, required a force that dislocated one hip, although the effort was made half an hour after death. He expired with symptoms of compression of the brain, on the evening of January 4th, 1848.

*Examination of the Brain, Eighteen Hours after Death*.—On removing the calvarium, the *dura mater* at one point was slightly torn by the tooth of the saw; from this opening 1½ or 2 ℥ of serous fluid escaped. On removing the *dura mater*, the surface of the brain was covered with a layer of coagulable lymph—the *pia mater* minutely and delicately injected. On cutting into the substance of the brain, it was found to retain all its original firmness. The suspicion had been entertained by the faculty at Utica and Hudson that there was extensive softening of the brain. A very minute examination disclosed nothing of the kind. While removing successive layers of the brain, each of which was carefully examined, we found in the centre of the cineritious portion of the cerebrum, a cyst, of the size of a small hazle nut, containing a semi-transparent fluid, which escaped, leaving only a delicate sac. On opening the ventricle of the right hemisphere we found it filled with serum, and on the floor of the ventricle was a mass of hydatids, held down by a fine net work of vessels, and a few delicate adhesions.

In the left ventricle there were, also, a quantity of serum, and a similar, but larger mass of hydatids, occupying the whole floor of the ventricle. The cerebellum was comparatively smaller than the cerebrum; its vessels, as also those of the upper portion of the spinal cord, (some three inches of which were removed with the cerebellum,) were minutely injected. On the lower portion of the cerebellum, posterior to the spinal chord, and within the *dura mater*, was found, what appeared originally to have been a coagulum, now perfectly organized, of the diameter of a shilling, its vessels anastomosing with those of the *dura mater*, and the base of the cerebellum.

The serum found effused in the ventricles and under the *dura mater*, was probably the sequel of a long continued state of congestion, traces of which were found in every portion of the brain, and the effusion the immediate cause of death.

But it may be a question for the curious, whether the hydatids were the primary cause of the dementia of the patient, or whether they, together with prostrated intellect, were a consequence of previous disease of the brain.

Lawrenceville, May 1, 1849.

